# Pyorrhœa Alveolaris

**Published:** 1899-07

**Authors:** Harold Cardwell

**Affiliations:** University of Pennsylvania


### PYORRHŒA ALVEOLARIS.

BY HAROLD CARDWELL, UNIVERSITY OF PENNSYLVANIA.

PATIENT, Mrs. B., aged fifty-one years, first came to Dental Hall on Wednesday, March 27, 1899. She complained of pain in her lower teeth when eating. Upon examination the six anterior lower teeth, the only remaining ones in her mouth, were found to be very loose, the gums red and inflamed, very large collections of tartar on the lingual aspects of the teeth. Slight exudation of pus from the sockets upon pressure of the gum tissue around the teeth. The patient was in a very bad state of health, having been under the care of a doctor for thirteen years. The case was diagnosed as one of pyorrhœa alveolaris.

*Treatment.*—The teeth were well scaled, and great care taken to remove all calculi. The sockets of the gums were well washed out with warm water. Hydrogen dioxide was then injected into them. After again washing out with warm water, sulphuric acid, fifty per cent., was gently placed around the teeth and in the pockets with a piece of orange-wood. The acid, after being left in for about a minute, was neutralized with sodium bicarbonate. The pockets were again washed out with warm water, and then well packed with quinine. The patient was told to use the following mouth-wash several times a day, and to keep the teeth clean:

℞ Hydronaphthol, gr. xv;

Alcoholis,

Aquæ destillatæ, āā ℥i. M.

Sig.—Put twenty drops of the above in a tumbler of water, and use as a wash.

Patient was again seen on April 2, 11, 18, and May 2. The pockets were all washed out each time with hydrogen dioxide, packed with quinine, leaving out the acid treatment. The teeth became very much firmer, and the patient had no longer pain on biting with them. The gums assumed their normal position, to a great extent, and seemed to be in a healthy state. Patient was dismissed, and told to keep on with the mouth-wash. The result was satisfactory, considering the age and condition of patient.

### Treatment of Pyorrhœa in Detail.

| Number. | Age. | History. | Number of Teeth. | Condition of Gingivæ. | Excretion. | Deposit. | Treatment. 1 | Treatment. 2 | Treatment. 3 | Treatment. 4 | Treatment. 5 | Treatment. 6 | Result. | Subsequent Complications. |
|---|---|---|---|---|---|---|---|---|---|---|---|---|---|---|
| 1 | 35 | None. | 10 | Hyperæmic. | Pus. | Calculi. | Scaled. | Hydrogen diox. | Sul. acid. | Soda bicarb. | Sul. quinine. | Hydronaphthol. | Cured. | None. |
| 2 | 59 | None. | ............... | Pockets. | None. | Calculi. | Scaled. | Hydrogen diox. | 20% sul. acid. | Soda bicarb. | Sul. quinine. | Hydronaphthol. | Cured. | None. |
| 3 | 47 | None. | 13 | Hyperæmic. | None. | Calculi. | Scaled. | Hydrogen diox. | 23% sul. acid. | Soda bicarb. | Sul. quinine. | Hydronaphthol. | Cured. | None. |
| 4 | 35 | None. | 32 | Teeth loose. | None. | Calculi. | Scaled. | Hydrogen diox. | 50% sul. acid. | Soda bicarb. | Sul. quinine. | Hydronaphthol. | Cured. | None. |
| 5 | 35 | None. | 3 | Spongy. | None. | Calculi. | Scaled. | Hydrogen diox. | 20% sul. acid. | Soda bicarb. | Sul. quinine. | Hydronaphthol. | Cured | None. |
| 6 | 35 | None. | 32 | Hyperæmic. | None. | None. | ............ | Hydrogen diox. | 20% sul. acid. | Soda bicarb. | Sul. quinine. | Hydronaphthol. | Cured. | None. |
| 7 | 35 | None. | 5 | Receded. | None. | Calculi. | Scaled. | Hydrogen diox. | 25% sul. acid. | Soda bicarb. | Sul. quinine. | Hydronaphthol. | Cured. | No report. |
| 8 | 30 | None. | 4 | Pockets. | None. | Calculi. | Scaled. | Hydrogen diox. | 25% sul. acid. | Soda bicarb. | Sul. quinine. | Hydronaphthol. | Cured. | None. |
| 9 | 41 | None. | All teeth. | Receded. | None. | Calculi. | Scaled. | Hydrogen diox. | 25% sul. acid. | Soda bicarb. | Sul. quinine. | Hydronaphthol. | Cured. | None. |
| 10 | 51 | Bad health. | 6 | No report. | None. | Calculi. | Scaled. | Hydrogen diox. | 50% sul. acid. | Soda bicarb. | Sul. quinine. | Hydronaphthol. | Cured. | None. |
| 11 | ... | None. | 14 | Teeth loose. | None. | Calculi. | Scaled. | Hydrogen diox. | 20% sul. acid. | Soda bicarb. | Sul. quinine. | Hydronaphthol. | Partially. | None. |
| 12 | 46 | None. | 4 | Teeth loose. | None. | On one tooth. | Scaled. | Hydrogen diox. | 25% sul. acid. | Soda bicarb. | Sul. quinine. | Hydronaphthol. | Cured. | None. |
| 13 | 42 | None. | 4 | Receded. | None. | Calculi. | Scaled. | Hydrogen diox. | 50% sul. acid. | Soda bicarb. | Sul. quinine. | Hydronaphthol. | Cured. | None. |
| 14 | 40 | None. | Several. | Teeth loose. | None. | Calculi. | Scaled. | Trichloracetic acid. | 25% sul. acid. | Soda bicarb. | Sul. quinine. | Hydronaphthol. | Cured. | None. |
| 15 | 40 | None. | All. | Receded, all teeth. | None. | Calculi. | Scaled. | Hydrogen diox. | 40% sul. acid. | Soda bicarb. | Sul. quinine. | Hydronaphthol. | Cured. | None. |
| 16 | 24 | Gout. | Lower teeth. | ..................... | None. | Calculi. | Scaled. | Hydrogen diox. | No report. | Soda bicarb. | Sul. quinine. | Hydronaphthol. | No result. | No report. |
| 17 | 30 | Gout. | All. | Receded. | None. | None. | ............ | Hydrogen diox. | 25% sul. acid. | Soda bicarb. | Protoiodide of mercury. | Hydronaphthol. | Cured. | None. |
| 18 | 39 | None. | 6 | Receded. | Pus. | Calculi. | Scaled. | Hydrogen diox. | 20% sul. acid. | Soda bicarb. | Sul. quinine. | Not used. | Partially. | No report. |
| 19 | 37 | None. | Several. | Receded. | Pus. | Calculi. | Scaled. | Hydrogen diox. | 20% sul. acid. | Soda bicarb. | Sul. quinine. | Hydronaphthol. | Cured. | None. |
| 20 | 20 | None. | Several. | Receded. | Pus. | Calculi. | Scaled. | Hydrogen diox. | 20% sul. acid. | Soda bicarb. | Sul. quinine. | Hydronaphthol. | Cured. | None. |
| 21 | 35 | Rheumatic. | All. | Receded. | Pus. | Calculi. | Scaled. | Hydrogen diox. | 20% sul. acid. | Soda bicarb. | Sul. quinine. | Hydronaphthol. | Partially. | No report. |
| 22 | 29 | None. | All. | Receded. | Pus. | Calculi. | Scaled. | Hydrogen diox. | 20% sul. acid. | Soda bicarb. | Sul. quinine. | Hydronaphthol. | Cured. | None. |
| 23 | ... | None. | All. | Receded. | Pus. | Calculi. | Scaled. | Hydrogen diox. | 25% sul. acid. | Soda bicarb. | Sul. quinine. | Hydronaphthol. | Cured. | None. |
| 24 | 45 | None. | 3 | Receded. | Pus. | Calculi. | Scaled. | Hydrogen diox. | 25% sul. acid. | Soda bicarb. | Sul. quinine. | Hydronaphthol. | Cured. | None. |
| 25 | ... | None. | All. | Hyperæmic. | Pus. | Calculi. | Scaled. | Hydrogen diox. | 25% sul. acid. | Soda bicarb. | Sul. quinine. | Hydronaphthol. | Cured. | None. |

REMARKS.

The foregoing presentation has been given for reasons stated (see note), and with the hope that some who have been at a loss in the treatment of these cases may be benefited by the enlarged experience made possible by a crowded infirmary.

It will be observed that in the tabulated cases that several have been complicated with systemic conditions supposed to be particularly antagonistic to local treatment,—rheumatism and gout. All of these have yielded to therapeutic treatment, in whole or in part. One of these was from the University Hospital, under care for this special complaint and thoroughly "saturated with uric acid," to use the expression of the medical attendant.

The pathological cases under care at the Dental Infirmary of the University, as a special test of the ability of the senior class, numbered one hundred and sixty-six, and of these, fifty cases of pyorrhœa were described. In the series of tabulated cases, twenty-five are alone given to avoid the monotony of repetition. Of the balance that were given the treatment described, all were satisfactorily cured, with the exception of three with no result stated, one still under care, and two partially helped.

It will be observed that in cases over sixty years of age the result was not entirely satisfactory. This goes very far to confirm my own view that pyorrhœa alveolaris must be separated into two stages, that occurring prior to that age and that presenting at a subsequent period. The first, according to this, should be classed as pyorrhœa proper, and the latter as senile pyorrhœa. From my observation, senile dentine, or, in other words, calcification of the tubulated structure, means a certain stoppage of nutrition. The tooth becomes a foreign body, and is not amenable to treatment. This must not be regarded as a mere hypothesis, but has been demonstrated by microscopic observation. It is generally manifested in old age, but may occur at a comparatively early period, as it has been observed by myself at thirty-five, and with the same unfortunate results as that following the later period.

The criticism may be made that this report has been prepared by undergraduates. While this is true, it does not lessen the value of the results. Great care was taken to have all pathological cases registered and watched by those in charge, and the largest proportion were seen by myself at various stages.

While it is true that the majority reported were treated after the method devised by myself, this was not made obligatory, in fact, was not desired, unless the student felt he could accomplish more by this than with other methods.

The plan generally adopted by the operators commended itself to me years ago, and has been repeatedly given to the dental profession, but from the continued complaints that it is impossible to cure pyorrhœa, it is presumed it has not been adopted, or, if used, has not been properly applied.

The use of sulphuric acid as an escharotic in these cases dates back many years. Who was the first to use it is unknown, but Dr. Atkinson certainly advocated it, and it may have originated in his fertile brain. The system adopted by myself included this, because of its positive value if applied properly. From writers upon this subject it is inferred that the aromatic sulphuric acid has been almost universally adopted. The use of this is believed to be a mistake. The strength, about twenty per cent., is not sufficient to accomplish desired results. Hence the commercial, pure as can be procured, is preferred, and reduced to twenty-five, thirty, or even fifty per cent., depending on the case to be treated. The length of time this is permitted to act upon the tissues is important. The usual period is *from one to two minutes,* and then it must be immediately neutralized by an antacid, sodium bicarbonate preferred. The escharotic, whether it be sulphuric acid, trichloracetic acid, or lactic acid, should not be repeated after the first application until at least ten days have elapsed. Time must be given for healthy granulations, and *these must not be disturbed.* If pus, after this period,—ten days,—should be present, the escharotic may be repeated, not otherwise.

The use of sulphate of quinine has not generally been understood. It has been in my hands, as a local application, one of the most valuable therapeutic agents; indeed, nothing that has been tried has supplanted it in the various gingival inflammations of the oral cavity. The peculiar properties of this agent have been fully explained elsewhere, and it is sufficient to say here that its topical effects have been tested to a degree that warrants its continued use. While satisfied with this agent, it is unquestionably true that there are several others that will answer, in part, the object to be attained by quinine in this disease. Very recently the protoiodide of mercury has been used with good results; one case will be found mentioned in the tabulated statement. It must be evident that the pockets must be closed to the ingress of pathogenic germs for a limited period to permit natural restoration of tissue, and this agent must be non-irritating and antiseptic in character.

The return of original conditions must be prevented by a continual use of an antiseptic mouth-wash. There is nothing in the whole range of antiseptics to be compared with beta-naphthol, or so-called hydronaphthol. With proper co-operation of the patient there need not be a recurrence of the disease. Indeed, I have never met with it where this care has been observed.

It may simply be a coincidence, but it is worthy of thought that, in one case described, the patient had suffered long from rheumatism, and was then under treatment by her physician for this disease. The treatment given not only effectually cured the pyorrhœa, but all symptoms of rheumatism disappeared, and the medical attendant was dismissed. This is a reversal of the theories held by those who explain pyorrhœa upon the uric acid hypothesis. Indeed, if these cases of rheumatism be accepted as cured by local treatment alone, the theoretical explanations alluded to will require modification, if they are not abandoned altogether.

A careful study of the cases described will enable those who may be interested to follow the course of treatment. *It must be exact to be successful.*

James Truman.

